# Crystal structure of ethyl 6-(chloro­meth­yl)-4-(4-chloro­phen­yl)-2-oxo-1,2,3,4-tetra­hydro­pyrimidine-5-carboxyl­ate

**DOI:** 10.1107/S1600536814023046

**Published:** 2014-10-24

**Authors:** S. Bharanidharan, H. Saleem, B. Gunasekaran, M. Syed Ali Padusha, M. Suresh

**Affiliations:** a Department of Physics, Annamalai University, Annamalainagar 608 002, Tamil Nadu, India; b Department of Physics & Nano Technology, SRM University, SRM Nagar, Kattankulathur, Kancheepuram District, Chennai 603 203, Tamil Nadu, India; c PG and Research Department of Chemistry, Jamal Mohamed College (Autonomous), Tiruchirappalli, Tamil Nadu 620 020, India; d PG and Research Dept of Chemistry, Jamal Mohamed College (Autonomous), Tiruchirappalli, Tamil Nadu 620 020, India

**Keywords:** crystal structure, tetra­hydro­pyrimidine, inversion dimers, anti­carcinogenic, anti­hypertensive, calcium channel modulators.

## Abstract

In the title compound, C_14_H_14_Cl_2_N_2_O_3_, the chloro­phenyl ring makes a dihedral angle of 87.08 (9)° with the tetra­hydro­pyrimidine ring. There is a short intra­molecular C—H⋯O contact present. In the crystal, mol­ecules are linked *via* pairs of N—H⋯O hydrogen bonds, forming inversion dimers with an *R* ^2^_2_(8) ring motif. The dimers are linked *via* a second pair of N—H⋯O hydrogen bonds, this time enclosing an *R* ^4^_4_(20) ring motif, forming ribbons along [100]. The ribbons are linked *via* C—H⋯O hydrogen bonds, forming sheets lying parallel to (001). The terminal ethyl group is disordered over two positions with an occupancy ratio of 0.654 (17):0.346 (17).

## Related literature   

For the many biological activities of di­hydro­pyrimidinone derivatives, see: Atwal *et al.* (1991 ▶); Jauk *et al.* (2000 ▶); Kato (1984 ▶); Wipf & Cunningham (1995 ▶); Bedia *et al.* (2006 ▶); For related structures, see: Nayak *et al.* (2009 ▶); Yuvaraj *et al.* (2010 ▶);
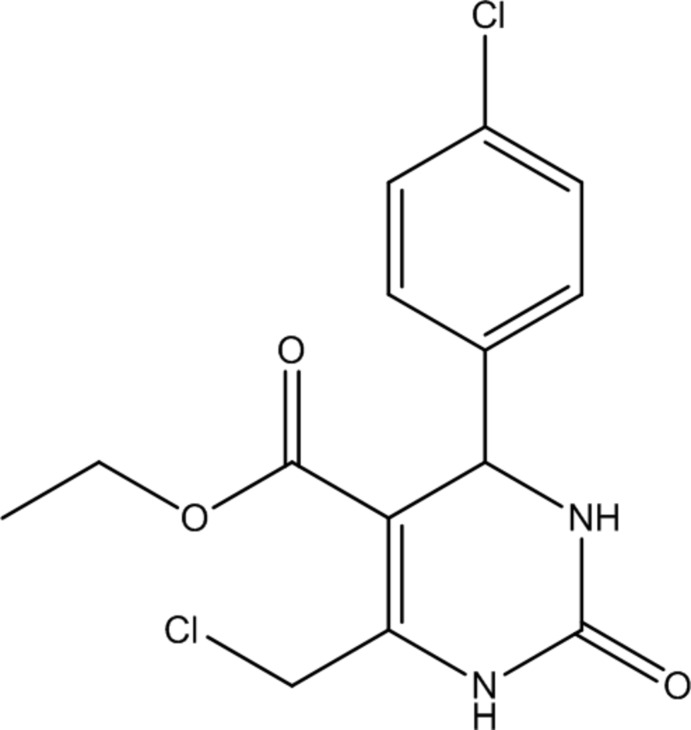


## Experimental   

### Crystal data  

C_14_H_14_Cl_2_N_2_O_3_
*M* *_r_* = 329.17
Triclinic, 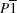

*a* = 7.4698 (3) Å
*b* = 9.1436 (3) Å
*c* = 12.6085 (4) Å
α = 107.147 (2)°
β = 99.941 (2)°
γ = 105.331 (2)°
*V* = 763.71 (5) Å^3^
*Z* = 2
Mo *K*α radiation
μ = 0.44 mm^−1^
*T* = 295 K
0.30 × 0.25 × 0.20 mm

### Data collection  

Bruker APEXII CCD diffractometer
Absorption correction: multi-scan (*SADABS*; Sheldrick, 1996 ▶) *T*_min_ = 0.897, *T*_max_ = 0.917
20157 measured reflections
3945 independent reflections
2911 reflections with *I* > 2σ(*I*)
*R*_int_ = 0.022

### Refinement  

*R*[*F* ^2^ > 2σ(*F* ^2^)] = 0.054
*wR*(*F* ^2^) = 0.153
*S* = 1.03
3945 reflections
211 parameters
26 restraints
H-atom parameters constrained
Δρ_max_ = 0.64 e Å^−3^
Δρ_min_ = −0.53 e Å^−3^

### 

Data collection: *APEX2* (Bruker, 2008 ▶); cell refinement: *SAINT* (Bruker, 2008 ▶); data reduction: *SAINT*; program(s) used to solve structure: *SHELXS97* (Sheldrick, 2008 ▶); program(s) used to refine structure: *SHELXL97* (Sheldrick, 2008 ▶); molecular graphics: *PLATON* (Spek, 2009 ▶); software used to prepare material for publication: *SHELXL97* and *PLATON*.

## Supplementary Material

Crystal structure: contains datablock(s) I. DOI: 10.1107/S1600536814023046/su5005sup1.cif

Structure factors: contains datablock(s) I. DOI: 10.1107/S1600536814023046/su5005Isup2.hkl

Supporting information file. DOI: 10.1107/S1600536814023046/su5005Isup3.cml

**Fig. 1.**
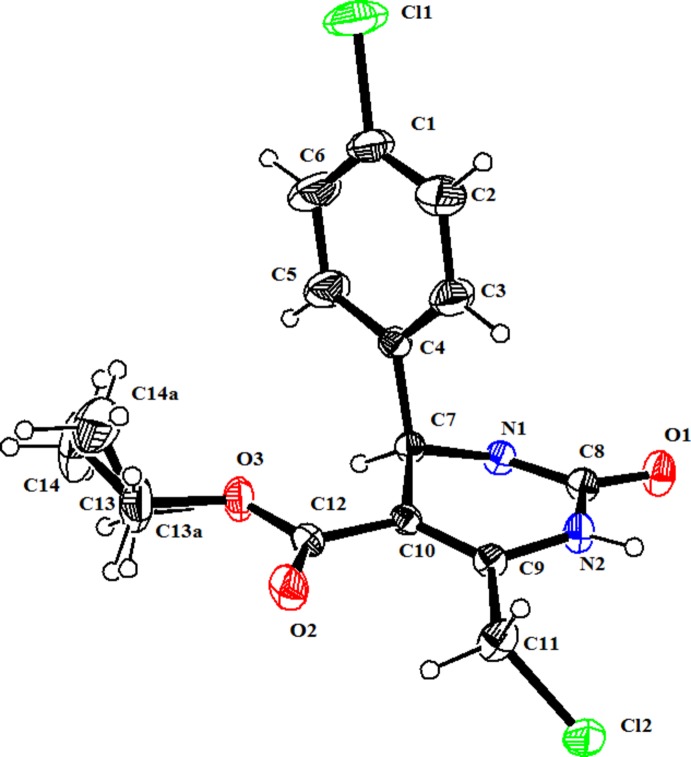
The molecular structure of the title molecule, with atom labelling. Displacement ellipsoids are drawn at the 30% probability level.

**Fig. 2.**
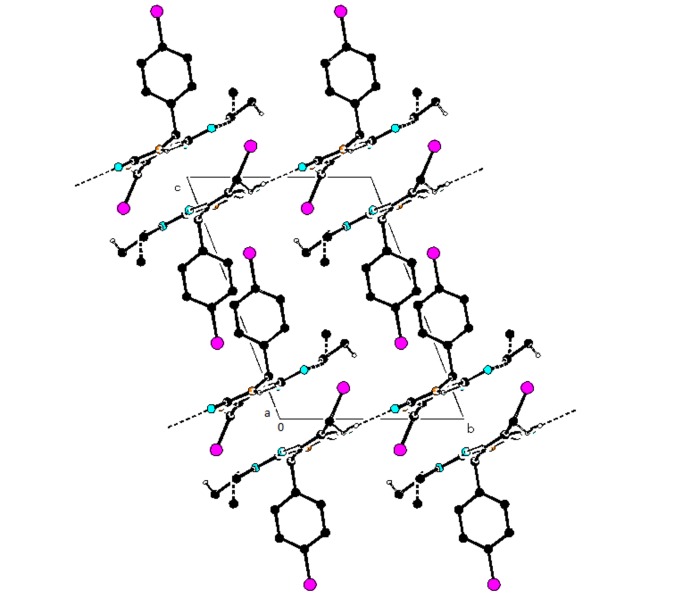
The crystal packing of the title compound, viewed along the a axis. Hydrogen bonds are shown as dashed lines (see Table 1 for details).

. DOI: 10.1107/S1600536814023046/su5005fig1.tif
The mol­ecular structure of the title mol­ecule, with atom labelling. Displacement ellipsoids are drawn at the 30% probability level.

a . DOI: 10.1107/S1600536814023046/su5005fig2.tif
The crystal packing of the title compound, viewed along the *a* axis. Hydrogen bonds are shown as dashed lines (see Table 1 for details).

CCDC reference: 1030125

Additional supporting information:  crystallographic information; 3D view; checkCIF report

## Figures and Tables

**Table 1:** Hydrogen-bond geometry (, )

*D*H*A*	*D*H	H*A*	*D* *A*	*D*H*A*
N2H2*A*O1^i^	0.86	2.04	2.885(2)	166
N1H1O2^ii^	0.86	2.23	3.070(2)	166
C11H11*B*O1^iii^	0.97	2.50	3.069(3)	117
C11H11*A*O2	0.97	2.14	2.814(3)	126

Symmetry codes: (i) 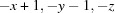; (ii) 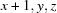; (iii) 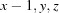.

## supplementary crystallographic information

### S1. Comment

Dihydropyrimidinones (DHPM) and their derivatives are important on account of their wide range of applications in therapeutic and pharmacology, namely because of their anticarcinogenic (Kato, 1984), antihypertensive (Atwal *et al.*, 1991) and calcium channel modulators (Jauk *et al.*, 2000) activities. These derivatives have also been screened for anti-bacterial (Wipf & Cunningham, 1995), and anti-tuberculosis activity (Bedia *et al.*, 2006).

The geometric parameters of the title molecule (Fig. 1) agree well with those reported fro similar structures (Nayak *et al.*, 2009; Yuvaraj *et al.*, 2010). The chlorophenyl ring makes a dihedral angles of 87.08 (9) ° with the tetrahydropyrimidine ring. There is a short intramolecular C—H···O contact (Table 1).

In the crystal, molecules are linked via a pair of N—H···O hydrogen bonds forming inversion dimers with an *R*^2^_2_(8) ring motif. The dimers are linked *via* a second pair of N—H···O hydrogen bonds, this time enclosing an *R*^4^_4_(20) ring motif, forming ribbons along [100]. The ribbons are linked *via* C—H···O hydrogen bonds forming sheets lying parallel to (001); see Table 1 and Fig. 2 for details.

### S2. Experimental

A mixture of ethyl-4-chloro acetoacetate (4.1 ml, 0.025 mol), 4-chlorobenzaldehyde (3.6 g, 0.025 mol), and urea (4.5 g, 0.075 mol) in ethanol (5 ml) was heated under reflux in the presence of concentrated HCl (1 mL) for 5 h (monitored by TLC). The reaction mixture, after being cooled to room temperature, was poured onto crushed ice and stirred for 5–10 min. The solid was separated and filtered under suction, washed with ice-cold water (50 ml), and then recrystallized from hot ethanol to afford pure product [m.p. 437 K; yield 76%].

### S3. Refinement

The terminal ethyl group is disordered over two position. The refined site occupancies of the disordered C atoms are C13/C14 = 0.654 (17) and C13A/C14A = 0.346 (17). The O3—C13A and C13A—C14A bond distances was restrained to be 1.400 (1) Å. In the refinement, ISOR was used for atoms C13, C14, C13A and C14A. The H atoms were positioned geometrically and refined using a riding model: N—H = 0.86 Å, C—H = 0.93 - 0.98 Å with U_iso_(H) = 1.5U_eq_(C) for methyl H atoms and = 1.2U_eq_(N,C) for other H atoms.

### Crystal data

**Table:**

C_14_H_14_Cl_2_N_2_O_3_	*Z* = 2
*M**_r_* = 329.17	*F*(000) = 340
Triclinic, *P*1	*D*_x_ = 1.431 Mg m^−^^3^
Hall symbol: -P 1	Mo *K*α radiation, λ = 0.71073 Å
*a* = 7.4698 (3) Å	Cell parameters from 4865 reflections
*b* = 9.1436 (3) Å	θ = 2.5–30.1°
*c* = 12.6085 (4) Å	µ = 0.44 mm^−^^1^
α = 107.147 (2)°	*T* = 295 K
β = 99.941 (2)°	Block, colourless
γ = 105.331 (2)°	0.30 × 0.25 × 0.20 mm
*V* = 763.71 (5) Å^3^	

### Data collection

**Table:**

Bruker APEXII CCD diffractometer	3945 independent reflections
Radiation source: fine-focus sealed tube	2911 reflections with *I* > 2σ(*I*)
Graphite monochromator	*R*_int_ = 0.022
Detector resolution: 0 pixels mm^-1^	θ_max_ = 31.0°, θ_min_ = 2.5°
ω and φ scans	*h* = −10→10
Absorption correction: multi-scan (*SADABS*; Sheldrick, 1996)	*k* = −12→12
*T*_min_ = 0.897, *T*_max_ = 0.917	*l* = −18→17
20157 measured reflections	

### Refinement

**Table:**

Refinement on *F*^2^	Primary atom site location: structure-invariant direct methods
Least-squares matrix: full	Secondary atom site location: difference Fourier map
*R*[*F*^2^ > 2σ(*F*^2^)] = 0.054	Hydrogen site location: inferred from neighbouring sites
*wR*(*F*^2^) = 0.153	H-atom parameters constrained
*S* = 1.03	*w* = 1/[σ^2^(*F*_o_^2^) + (0.0628*P*)^2^ + 0.6453*P*] where *P* = (*F*_o_^2^ + 2*F*_c_^2^)/3
3945 reflections	(Δ/σ)_max_ < 0.001
211 parameters	Δρ_max_ = 0.64 e Å^−^^3^
26 restraints	Δρ_min_ = −0.53 e Å^−^^3^

### Fractional atomic coordinates and isotropic or equivalent isotropic displacement parameters (Å^2^)

**Table:**

	*x*	*y*	*z*	*U*_iso_*/*U*_eq_	Occ. (<1)
C1	0.8422 (5)	0.1384 (4)	0.5387 (2)	0.0651 (8)	
C2	0.7145 (5)	−0.0099 (4)	0.4712 (3)	0.0725 (9)	
H2	0.6712	−0.0878	0.5031	0.087*	
C3	0.6488 (4)	−0.0442 (3)	0.3538 (2)	0.0598 (7)	
H3	0.5588	−0.1453	0.3073	0.072*	
C4	0.7137 (3)	0.0674 (3)	0.30506 (18)	0.0361 (4)	
C5	0.8424 (5)	0.2165 (4)	0.3761 (3)	0.0699 (9)	
H5	0.8876	0.2950	0.3452	0.084*	
C6	0.9060 (6)	0.2518 (4)	0.4938 (3)	0.0895 (12)	
H6	0.9925	0.3537	0.5414	0.107*	
C7	0.6508 (3)	0.0279 (2)	0.17587 (17)	0.0306 (4)	
H7	0.7032	0.1280	0.1613	0.037*	
C8	0.6382 (3)	−0.2509 (2)	0.06968 (18)	0.0342 (4)	
C9	0.3408 (3)	−0.1913 (2)	0.06544 (17)	0.0315 (4)	
C10	0.4341 (3)	−0.0329 (2)	0.12644 (16)	0.0300 (4)	
C11	0.1275 (3)	−0.2650 (3)	0.0111 (2)	0.0454 (5)	
H11A	0.0752	−0.1799	0.0068	0.055*	
H11B	0.0673	−0.3162	0.0593	0.055*	
C12	0.3328 (3)	0.0848 (3)	0.15276 (18)	0.0360 (4)	
N1	0.7306 (2)	−0.0910 (2)	0.11509 (15)	0.0338 (4)	
H1	0.8450	−0.0555	0.1081	0.041*	
N2	0.4415 (2)	−0.2989 (2)	0.05067 (17)	0.0375 (4)	
H2A	0.3781	−0.4009	0.0285	0.045*	
O1	0.7187 (2)	−0.35121 (19)	0.04214 (16)	0.0488 (4)	
O2	0.1610 (2)	0.0561 (2)	0.13615 (16)	0.0508 (4)	
O3	0.4586 (2)	0.23490 (19)	0.20251 (17)	0.0554 (5)	
C13	0.374 (3)	0.3618 (16)	0.2478 (12)	0.086 (4)	0.654 (17)
H13A	0.2924	0.3745	0.1847	0.103*	0.654 (17)
H13B	0.2959	0.3318	0.2971	0.103*	0.654 (17)
C14	0.5303 (12)	0.5098 (7)	0.3121 (11)	0.116 (4)	0.654 (17)
H14A	0.6081	0.4964	0.3752	0.174*	0.654 (17)
H14B	0.4803	0.5955	0.3415	0.174*	0.654 (17)
H14C	0.6076	0.5371	0.2627	0.174*	0.654 (17)
C13A	0.394 (4)	0.368 (3)	0.2319 (11)	0.070 (7)	0.346 (17)
H13C	0.2598	0.3357	0.1900	0.084*	0.346 (17)
H13D	0.4684	0.4531	0.2098	0.084*	0.346 (17)
C14A	0.414 (4)	0.428 (3)	0.3506 (12)	0.127 (10)	0.346 (17)
H14D	0.3199	0.3533	0.3697	0.190*	0.346 (17)
H14E	0.3937	0.5314	0.3713	0.190*	0.346 (17)
H14F	0.5409	0.4415	0.3920	0.190*	0.346 (17)
Cl1	0.92366 (19)	0.18204 (17)	0.68608 (7)	0.1114 (4)	
Cl2	0.07043 (8)	−0.41074 (8)	−0.12863 (5)	0.0522 (2)	

### Atomic displacement parameters (Å^2^)

**Table:**

	*U*^11^	*U*^22^	*U*^33^	*U*^12^	*U*^13^	*U*^23^
C1	0.0719 (18)	0.085 (2)	0.0334 (12)	0.0407 (17)	0.0004 (12)	0.0089 (13)
C2	0.095 (2)	0.079 (2)	0.0475 (16)	0.0299 (19)	0.0186 (15)	0.0296 (15)
C3	0.0751 (18)	0.0529 (15)	0.0402 (13)	0.0083 (13)	0.0088 (12)	0.0163 (11)
C4	0.0315 (10)	0.0396 (11)	0.0348 (10)	0.0151 (8)	0.0040 (8)	0.0098 (8)
C5	0.076 (2)	0.0498 (15)	0.0523 (16)	−0.0029 (14)	−0.0110 (14)	0.0120 (12)
C6	0.099 (3)	0.069 (2)	0.0506 (18)	0.0008 (19)	−0.0216 (17)	−0.0003 (16)
C7	0.0246 (9)	0.0292 (9)	0.0363 (10)	0.0085 (7)	0.0058 (7)	0.0110 (8)
C8	0.0261 (9)	0.0363 (10)	0.0398 (10)	0.0112 (8)	0.0095 (8)	0.0122 (8)
C9	0.0228 (8)	0.0364 (10)	0.0361 (10)	0.0107 (7)	0.0095 (7)	0.0124 (8)
C10	0.0255 (9)	0.0331 (9)	0.0325 (9)	0.0112 (7)	0.0066 (7)	0.0126 (8)
C11	0.0262 (10)	0.0453 (12)	0.0516 (13)	0.0079 (9)	0.0099 (9)	0.0027 (10)
C12	0.0343 (10)	0.0374 (10)	0.0370 (10)	0.0163 (8)	0.0061 (8)	0.0123 (8)
N1	0.0207 (7)	0.0351 (9)	0.0416 (9)	0.0079 (6)	0.0086 (6)	0.0094 (7)
N2	0.0237 (8)	0.0284 (8)	0.0552 (11)	0.0069 (6)	0.0101 (7)	0.0096 (7)
O1	0.0303 (8)	0.0385 (8)	0.0734 (12)	0.0151 (6)	0.0150 (7)	0.0103 (8)
O2	0.0333 (8)	0.0521 (10)	0.0681 (11)	0.0229 (7)	0.0135 (7)	0.0155 (8)
O3	0.0431 (9)	0.0336 (8)	0.0738 (12)	0.0180 (7)	−0.0026 (8)	0.0034 (8)
C13	0.071 (6)	0.051 (6)	0.116 (9)	0.039 (4)	0.008 (6)	−0.001 (5)
C14	0.100 (5)	0.041 (3)	0.171 (9)	0.017 (3)	0.030 (5)	−0.003 (4)
C13A	0.077 (14)	0.052 (10)	0.063 (8)	0.048 (10)	−0.016 (7)	−0.009 (7)
C14A	0.18 (2)	0.138 (18)	0.075 (9)	0.115 (18)	0.028 (9)	0.000 (9)
Cl1	0.1383 (10)	0.1531 (11)	0.0355 (4)	0.0742 (8)	0.0001 (5)	0.0144 (5)
Cl2	0.0426 (3)	0.0510 (4)	0.0510 (3)	0.0153 (3)	−0.0023 (2)	0.0100 (3)

### Geometric parameters (Å, º)

**Table:**

C1—C6	1.342 (5)	C11—Cl2	1.766 (2)
C1—C2	1.352 (5)	C11—H11A	0.9700
C1—Cl1	1.739 (3)	C11—H11B	0.9700
C2—C3	1.388 (4)	C12—O2	1.207 (3)
C2—H2	0.9300	C12—O3	1.330 (3)
C3—C4	1.366 (3)	N1—H1	0.8600
C3—H3	0.9300	N2—H2A	0.8600
C4—C5	1.369 (3)	O3—C13A	1.4000 (10)
C4—C7	1.518 (3)	O3—C13	1.482 (8)
C5—C6	1.387 (4)	C13—C14	1.430 (19)
C5—H5	0.9300	C13—H13A	0.9700
C6—H6	0.9300	C13—H13B	0.9700
C7—N1	1.461 (2)	C14—H14A	0.9600
C7—C10	1.513 (2)	C14—H14B	0.9600
C7—H7	0.9800	C14—H14C	0.9600
C8—O1	1.225 (2)	C13A—C14A	1.4000 (10)
C8—N1	1.333 (3)	C13A—H13C	0.9700
C8—N2	1.373 (2)	C13A—H13D	0.9700
C9—C10	1.341 (3)	C14A—H14D	0.9600
C9—N2	1.379 (2)	C14A—H14E	0.9600
C9—C11	1.498 (3)	C14A—H14F	0.9600
C10—C12	1.466 (3)		
			
C6—C1—C2	121.0 (3)	Cl2—C11—H11A	109.2
C6—C1—Cl1	119.8 (3)	C9—C11—H11B	109.2
C2—C1—Cl1	119.2 (3)	Cl2—C11—H11B	109.2
C1—C2—C3	119.1 (3)	H11A—C11—H11B	107.9
C1—C2—H2	120.5	O2—C12—O3	122.43 (19)
C3—C2—H2	120.5	O2—C12—C10	127.3 (2)
C4—C3—C2	121.3 (3)	O3—C12—C10	110.28 (17)
C4—C3—H3	119.4	C8—N1—C7	124.46 (16)
C2—C3—H3	119.4	C8—N1—H1	117.8
C3—C4—C5	118.0 (2)	C7—N1—H1	117.8
C3—C4—C7	121.6 (2)	C8—N2—C9	123.10 (17)
C5—C4—C7	120.4 (2)	C8—N2—H2A	118.5
C4—C5—C6	120.8 (3)	C9—N2—H2A	118.4
C4—C5—H5	119.6	C12—O3—C13A	120.4 (14)
C6—C5—H5	119.6	C12—O3—C13	114.8 (8)
C1—C6—C5	119.8 (3)	C13A—O3—C13	10.8 (18)
C1—C6—H6	120.1	C14—C13—O3	107.3 (12)
C5—C6—H6	120.1	C14—C13—H13A	110.3
N1—C7—C10	109.45 (15)	O3—C13—H13A	110.3
N1—C7—C4	110.73 (15)	C14—C13—H13B	110.3
C10—C7—C4	113.47 (16)	O3—C13—H13B	110.3
N1—C7—H7	107.7	H13A—C13—H13B	108.5
C10—C7—H7	107.7	O3—C13A—C14A	110.7 (9)
C4—C7—H7	107.7	O3—C13A—H13C	109.5
O1—C8—N1	123.56 (18)	C14A—C13A—H13C	109.5
O1—C8—N2	120.66 (19)	O3—C13A—H13D	109.5
N1—C8—N2	115.73 (17)	C14A—C13A—H13D	109.5
C10—C9—N2	120.01 (17)	H13C—C13A—H13D	108.1
C10—C9—C11	124.57 (18)	C13A—C14A—H14D	109.5
N2—C9—C11	115.41 (18)	C13A—C14A—H14E	109.5
C9—C10—C12	122.35 (17)	H14D—C14A—H14E	109.5
C9—C10—C7	119.80 (17)	C13A—C14A—H14F	109.5
C12—C10—C7	117.78 (17)	H14D—C14A—H14F	109.5
C9—C11—Cl2	112.02 (15)	H14E—C14A—H14F	109.5
C9—C11—H11A	109.2		
			
C6—C1—C2—C3	−0.1 (5)	C10—C9—C11—Cl2	138.10 (19)
Cl1—C1—C2—C3	179.6 (3)	N2—C9—C11—Cl2	−42.8 (3)
C1—C2—C3—C4	1.4 (5)	C9—C10—C12—O2	8.7 (4)
C2—C3—C4—C5	−1.7 (5)	C7—C10—C12—O2	−168.2 (2)
C2—C3—C4—C7	176.7 (3)	C9—C10—C12—O3	−173.0 (2)
C3—C4—C5—C6	0.7 (5)	C7—C10—C12—O3	10.1 (3)
C7—C4—C5—C6	−177.7 (3)	O1—C8—N1—C7	−163.2 (2)
C2—C1—C6—C5	−0.8 (6)	N2—C8—N1—C7	19.2 (3)
Cl1—C1—C6—C5	179.4 (3)	C10—C7—N1—C8	−31.1 (3)
C4—C5—C6—C1	0.5 (6)	C4—C7—N1—C8	94.8 (2)
C3—C4—C7—N1	−68.6 (3)	O1—C8—N2—C9	−171.1 (2)
C5—C4—C7—N1	109.8 (3)	N1—C8—N2—C9	6.6 (3)
C3—C4—C7—C10	55.0 (3)	C10—C9—N2—C8	−16.8 (3)
C5—C4—C7—C10	−126.6 (3)	C11—C9—N2—C8	164.1 (2)
N2—C9—C10—C12	−174.98 (18)	O2—C12—O3—C13A	−3.8 (9)
C11—C9—C10—C12	4.0 (3)	C10—C12—O3—C13A	177.8 (9)
N2—C9—C10—C7	1.8 (3)	O2—C12—O3—C13	6.6 (7)
C11—C9—C10—C7	−179.16 (19)	C10—C12—O3—C13	−171.8 (7)
N1—C7—C10—C9	19.3 (3)	C12—O3—C13—C14	171.9 (10)
C4—C7—C10—C9	−105.0 (2)	C13A—O3—C13—C14	−65 (9)
N1—C7—C10—C12	−163.80 (17)	C12—O3—C13A—C14A	103 (2)
C4—C7—C10—C12	72.0 (2)	C13—O3—C13A—C14A	42 (7)

### Hydrogen-bond geometry (Å, º)

**Table:**

*D*—H···*A*	*D*—H	H···*A*	*D*···*A*	*D*—H···*A*
N2—H2*A*···O1^i^	0.86	2.04	2.885 (2)	166
N1—H1···O2^ii^	0.86	2.23	3.070 (2)	166
C11—H11*B*···O1^iii^	0.97	2.50	3.069 (3)	117
C11—H11*A*···O2	0.97	2.14	2.814 (3)	126

Symmetry codes: (i) −*x*+1, −*y*−1, −*z*; (ii) *x*+1, *y*, *z*; (iii) *x*−1, *y*, *z*.
