# Supplementary figures and images for: Crystal structure of ethyl 6-(chloro­meth­yl)-4-(4-chloro­phen­yl)-2-oxo-1,2,3,4-tetra­hydro­pyrimidine-5-carboxyl­ate
Source: Acta Crystallogr Sect E Struct Rep Online. 2014 Oct 24;70(Pt 11):o1185–6. doi: 10.1107/S1600536814023046 (PMC4257316; doi:10.1107/S1600536814023046)

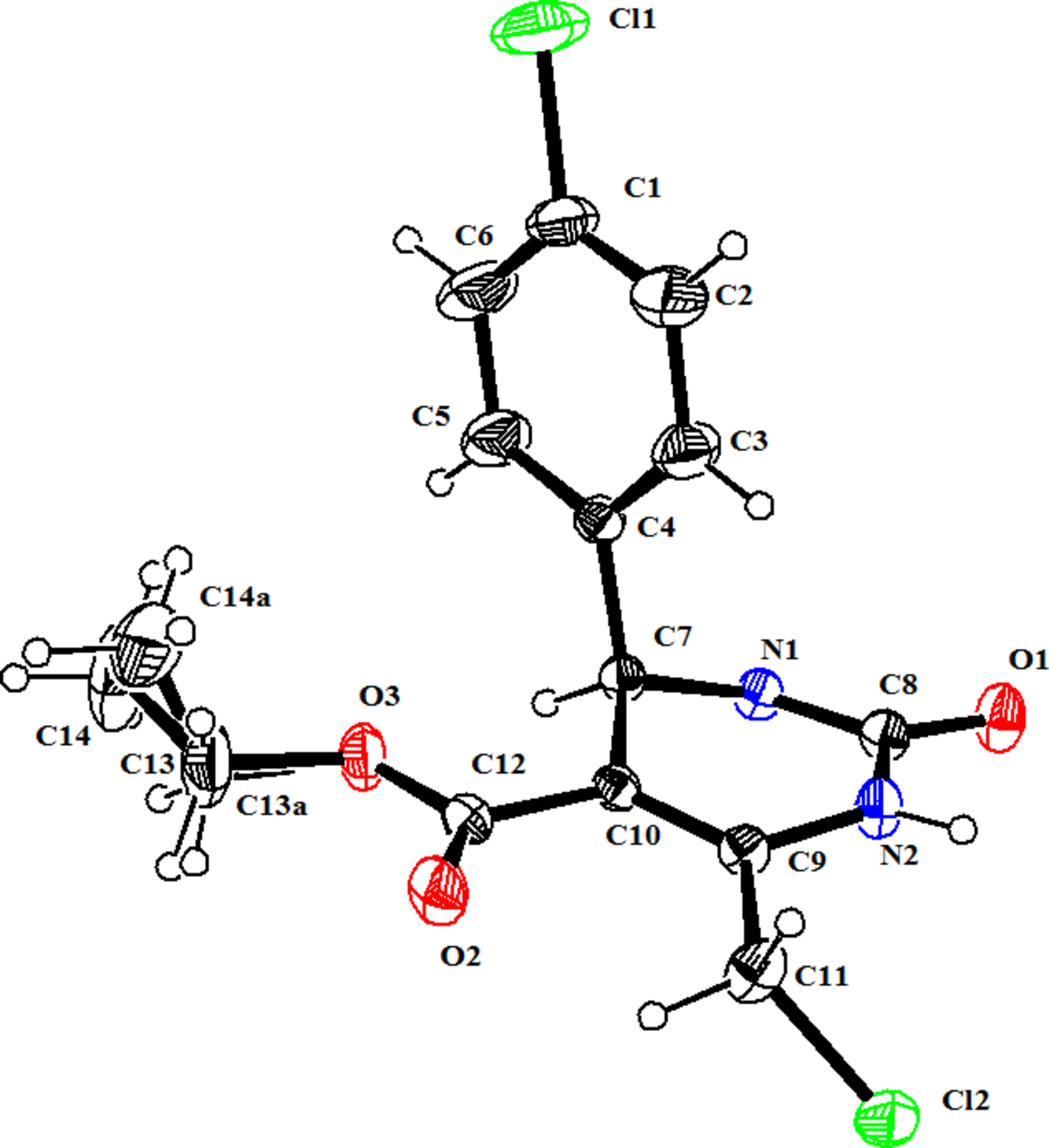

Supplement: Supplementary file 4 [file e-70-o1185-fig1.tif]

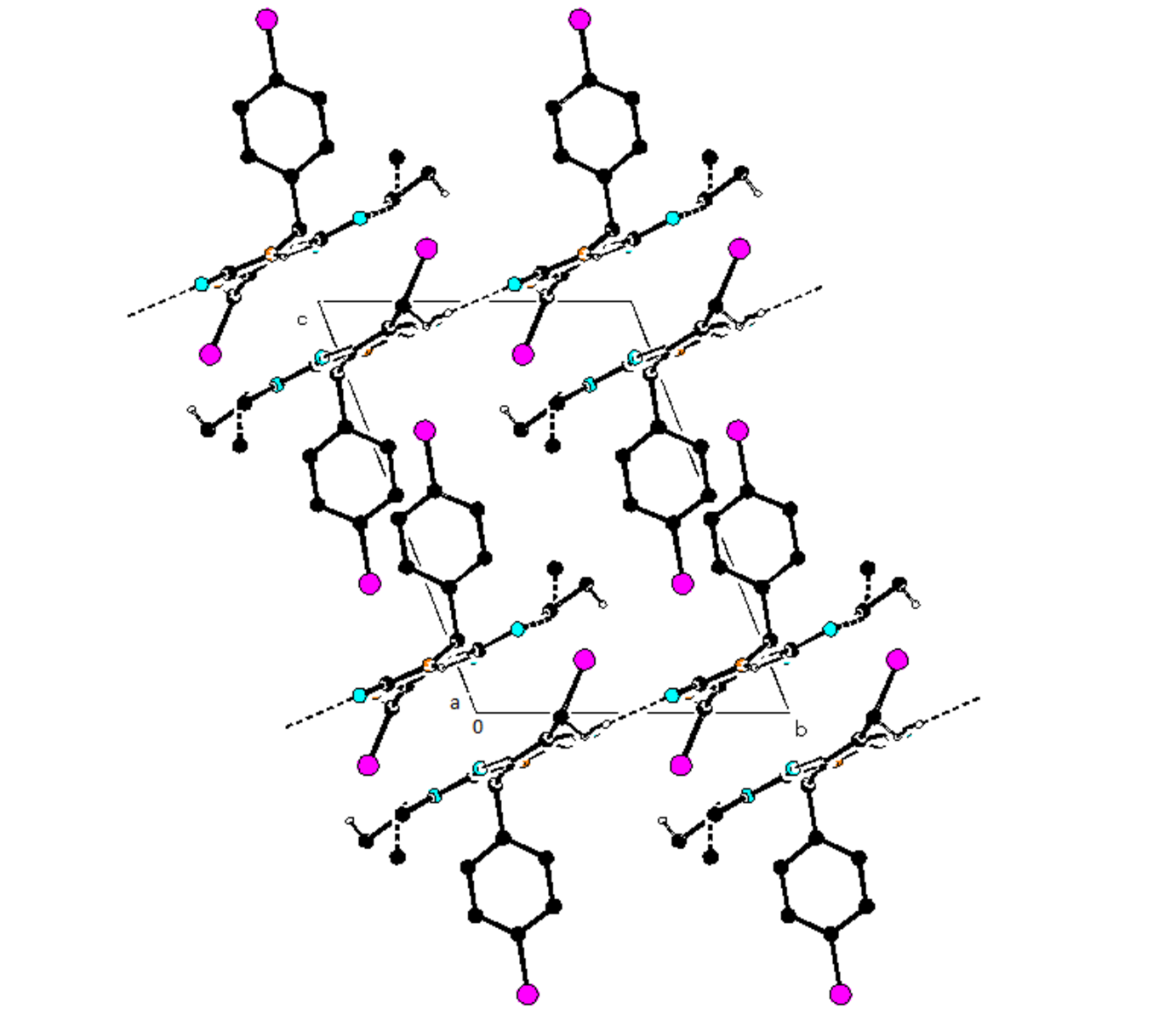

Supplement: Supplementary file 5 [file e-70-o1185-fig2.tif]
